# P-1857. S100B and other Fecal Biomarkers as Predictors of *Clostridioides difficile* Infection Severity and Recurrence are Influenced by Age

**DOI:** 10.1093/ofid/ofae631.2018

**Published:** 2025-01-29

**Authors:** Laasya Doppalapudi, Matthew Behm, Deiziane Costa, Mônica Rodrigues-Jesus, Jae Hyun Shin, Cirle A Warren

**Affiliations:** University of Virginia Division of Infectious Diseases and International Health, Charlottesville, Virginia; University of Virginia Division of Infectious Diseases and International Health, Charlottesville, Virginia; University of Virginia, Charlottesville, Virginia; University of Virginia Division of Infectious Diseases and International Health, Charlottesville, Virginia; University of Virginia, Charlottesville, Virginia; University of Virginia, Charlottesville, Virginia

## Abstract

**Background:**

Clostridioides difficile infection (CDI) is an urgent threat in the US. Because disease severity is strongly correlated with intestinal inflammation, the aim of this study was to identify fecal biomarkers associated with poor outcomes and investigate how these biomarkers are influenced by clinical variables.

**Methods:**

Fecal samples from patients diagnosed with CDI were collected from April 2021 to February 2022. Levels of biomarkers for intestinal inflammation (myeloperoxidase-MPO, lipocalin-2-Lcn2, calprotectin, S100 calcium-binding protein B-S100B, IL-6 and brain-derived neurotrophic factor-BDNF) were measured by ELISA. *C. difficile* isolates were tested for binary toxins gene, *cdtA/cdtB*, by qPCR.

**Results:**

The majority of patients were females (54%, 88/149), infected by binary positive strain (59%, 88/149) and with mean age of 61.4, (range: 21 to 96 years old). 43.2% (64/148) of the patients exhibited severe disease, 29.7% (44/148) were recurrent CDI cases, and 19.4% (29/149) died post-infection. Increased fecal levels of S100B (p=0.0006) and IL-6 (p=0.03) were detected in severe disease. Compared to non-severe cases, severe first CDI cases exhibited higher levels of S100B (p=0.002), and IL-6 (p=0.04), while severe recurrent cases showed significantly higher levels of MPO, Lcn2, and calprotectin. In addition, increased levels of MPO, calprotectin, and S100B correlated positively with age. Increased fecal IL-6 (p=0.03) and S100B (p=0.01) were detected in middle-aged (40-64 years old) severe cases. Increased MPO (p=0.02), calprotectin (p=0.01), Lcn2 (p=0.006), and S100B (p=0.02) were also detected in middle-aged and oldest-old severe cases. Furthermore, patients infected by binary positive strains had significantly higher levels of fecal MPO, Lcn2, calprotectin, and IL-6. Recurrent cases also showed higher levels of fecal MPO, Lcn2, and calprotectin compared to first episode CDI cases. Fecal markers of inflammation were independent of gender.

**Conclusion:**

Our findings indicate that fecal S100B is a marker of CDI severity in middle-aged, middle-old, and oldest-old patients independent of gender. Additionally, elevated fecal biomarkers (MPO, Lcn2, calprotectin, S100B, and IL-6) appear to correlate with binary toxin positivity and recurrence.

**Disclosures:**

Jae Hyun Shin, MD, Ferring: Grant/Research Support Cirle A. Warren, MD, Ferring Pharmaceuticals: Site PI for ROAR

